# 吉非替尼耐药的人肺腺癌HCC-827/GR细胞乙醛脱氢酶亚型表达分析

**DOI:** 10.3779/j.issn.1009-3419.2018.06.01

**Published:** 2018-06-20

**Authors:** 婷婷 杨, 晶晶 古, 婷 刘, 海滨 马, 晓娜 马, 金 陶, 毅然 金, 雪云 梁

**Affiliations:** 1 750004 银川，宁夏医科大学总医院，宁夏人类干细胞研究所 Institute of Human Stem Cell Research, Yinchuan 750004, China; 2 750004 银川，宁夏医科大学临床医学院 College of Clinical Medicine, General Hospital of Ningxia Medical University, Yinchuan 750004, China

**Keywords:** 人肺腺癌, HCC-827, 吉非替尼, 乙醛脱氢酶, Human lung adenocarcinoma, HCC-827, Gefitinib, Acetaldehyde dehydrogenase

## Abstract

**背景与目的:**

肿瘤的复发和耐药是肿瘤患者死亡的主要原因。乙醛脱氢酶（acetaldehyde dehydrogenase, ALDH）家族与肿瘤细胞的增殖、迁移、侵袭和耐药密切相关，且ALDH不同亚型基因在不同肿瘤细胞中有差异性表达。本实验旨在分析对吉非替尼耐药的人肺腺癌细胞HCC-827/GR ALDH亚型的表达。

**方法:**

利用人肺腺癌细胞系HCC-827制备吉非替尼耐药细胞株HCC-827/GR；利用流式检测HCC-827及HCC-827/GR中ALDH的表达；采用MTT法检测ALDH抑制剂二乙氨基苯甲醛（diethyllaminaldehyde, DEAB）处理前后HCC-827/GR细胞的增殖能力和对吉非替尼的敏感性；利用qRT-PCR检测HCC-827与HCC-827/GR细胞中ALDH各亚型在mRNA水平的表达。

**结果:**

与HCC-827细胞相比，ALDH在吉非替尼耐药的细胞株HCC-827/GR的阳性率增加；经100 μmol/L DEAB处理后，HCC-827/GR细胞增殖能力下降；与HCC-827细胞相比，ALDH1A1和ALDH1L1在HCC-827/GR细胞中mRNA表达水平增高；ALDH3B2表达降低。

**结论:**

ALDH具有检测吉非替尼耐药的人肺腺癌细胞的标志分子的潜力，其中ALDH1A1可能参与人肺腺癌细胞对吉非替尼耐药性的形成过程。

## Abstract (en)

**Background and objective:**

Tumor recurrence and drug resistance are the main causes of death in tumor patients. The family of acetaldehyde dehydrogenase (ALDH) is closely related to the proliferation, migration, invasion and resistance of tumor cells, and different ALDH subtypes are expressed in different tumor cells. The aim of this study is to elucidate the ALDH subtype in human lung adenocarcinoma HCC-827/GR cells, which resistant to the gefitinib.

**Methods:**

The human lung adenocarcinoma HCC-827 cells were used to generate the gefitinib-resistant HCC-827/GR cells; the expression of ALDH subtype in either HCC-827 or HCC-827/GR was detected by flow cytometry; The proliferative capacity and sensitivity to gefitinib of hcc-827/GR cells were analyzed by MTT assay before and after treatment with 100 μmol/L diethyllaminaldehyde (DEAB); Real-time quantitative PCR was used to detect the expression of ALDH subtypes at mRNA levels in hcc-827 cells and hcc-827/GR cells.

**Results:**

Compared with HCC-827 cells, the positive rate of ALDH in HCC-827/GR cells increased. The proliferation ability of HCC-827/GR cells decreased after treatment with 100 μmol/L DEAB. Compared with HCC-827 cells, the expression of ALDH1A1 and ALDH1L1 mRNA was increased in hcc-827/GR cells, but the ALDH3B2 expression was decreased.

**Conclusion:**

ALDH might be used as a molecular biomarker to test the gefitinib-resistant to lung adenocarcinoma cancer cells, and the ALDH1A1 may play a role in gefitinib resistance in lung cancer.

肿瘤的复发和耐药是肿瘤患者死亡的主要原因。肿瘤干细胞在肿瘤的发生、维持和转移中扮演了重要的角色。乙醛脱氢酶（ALDH）属于氧化还原酶类，对生物具有细胞毒性，致突变性，遗传毒性以及致癌性等性质^[1]^。ALDH为肿瘤干细胞的分子标志之一，已有研究证明，ALDH家族与肿瘤细胞的增殖、迁移、侵袭和耐药密切相关^[2]^，且ALDH不同亚型基因在不同肿瘤细胞中有差异性表达^[3]^，但是ALDH不同亚型在不同肿瘤细胞中的作用及机制，目前尚未有明确的研究结论。肺癌是全世界发病率最高的恶性肿瘤，临床上常见患者对吉非替尼化疗产生耐药性，本研究拟通过比较人肺腺癌细胞系及其吉非替尼耐药细胞中ALDH亚型基因表达，分析ALDH不同亚型在人肺腺癌细胞系及其吉非替尼耐药细胞中的差异，探讨ALDH在吉非替尼耐药的人肺腺癌HCC-827/GR细胞中的作用，为临床治疗吉非替尼耐药的肺腺癌提供新思路和方法。

## 1 材料与方法

### 1.1 材料

HCC-827人肺腺癌细胞株购自中南大学湘雅医学院细胞中心; RPMI-1640培养基和胎牛血清购自Gibico公司; ALDEFLUOR试剂盒购自Stem Cell公司; ALDEFLUOR试剂盒购自Stem Cell公司; 二乙基氨基苯甲醛（diethylaminobenzaldehyde, DEAB）固体结晶、实时定量PCR（real time quantitative PCR, qRT-PCR）试剂盒均购自Sigma公司; RNA提取试剂盒购自北京全式金生物技术有限公司; 逆转录试剂盒购自QIAGEN公司; 引物由生工生物工程（上海）有限公司合成。

### 1.2 方法

#### 1.2.1 实验分组

对照组为正常培养的人肺腺癌细胞株HCC-827;实验组为经梯度浓度筛选培养HCC-827细胞制备的对吉非替尼耐药细胞HCC-827/GR。

#### 1.2.2 细胞培养及吉非替尼耐药细胞HCC-827/GR筛选

用含100 mL/L胎牛血清的RPMI-1640培养基，37 ℃、5%CO_2_的培养条件正常培养HCC-827细胞，待细胞汇合至80%左右，加入0.1 μmol/mL吉非替尼，24 h后细胞发生大量死亡，仅有不到10%左右的细胞存活。将存活细胞继续在含0.1 μmol/mL吉非替尼培养基中培养，直至细胞密度达80%后传代。依此方法，将细胞培养液中吉非替尼浓度从0.1 μmol/mL、0.2 μmol/mL、0.4 μmol/mL、0.8 μmol/mL、1.0 μmol/mL依次逐级增加处理HCC-827细胞。当培养基中吉非替尼浓度达到1.0 μmol/mL后，细胞生长保持稳定状态，无大批细胞死亡。此时的细胞为HCC-827/GR细胞。

#### 1.2.3 流式细胞术检测细胞ALDH的表达

采用流式细胞仪对HCC-827及HCC-827/GR细胞中ALDH阳性细胞计数。具体操作依照ALDEFLUOR试剂盒说明书，每组细胞数量均为1×10^6^个，均以300 μL的PBS重悬。实验采用加入ALDH抑制剂DEAB的细胞ALDH表达作为阴性对照。

#### 1.2.4 吉非替尼和DEAB对人肺腺癌细胞HCC-827及其耐药株HCC-827/GR的毒性实验

采用MTT法，取对数生长期的HCC-827及HCC-827/GR制成5×10^4^/mL的细胞悬液，96孔板每孔接种200 μL。处理组1采用浓度为0、1 μmol/mL、5 μmol/mL、10 μmol/mL吉非替尼处理细胞，处理组2采用浓度为0、100 μmol/L、300 μmol/L、500 μmol /L DEAB处理细胞。置37 ℃、5%CO_2_培养箱培养72 h，每孔加MTT液20 μL，37 ℃孵育4 h; 吸取上清，每孔加入150 μL DMSO，振荡10 s使结晶充分溶解; 在490 nm处测定每孔的吸光度（*A*）值，计算细胞生长抑制率。

#### 1.2.5 检测吉非替尼和DEAB对耐药株HCC-827/GR增殖能力影响

采用MTT法（同上），根据细胞毒性实验结果，选取无细胞毒剂量1 μmol/mL吉非替尼和200 μmol/L DEAB进行实验，取对数生长期的HCC-827及HCC-827/GR细胞将其制成细胞数为5×10^4^/mL的细胞悬液，分别接种于96孔板每孔100 μL。24 h后配制含1 μmol/mL吉非替尼的RPMI-1640培养液，实验组弃上清每孔各加100 μL含药培养基，对照组不加; 6 h后添加含200 μmol/L DEAB的RPMI-1640培养液，实验组每孔各加100 μL（稀释后DEAB浓度为100 μmol/L），对照组加入100 μL正常培养基。于加药后24 h、48 h、72 h、96 h分别测定DEAB处理前后HCC-827/GR细胞增殖能力影响。

#### 1.2.6 qRT-PCR检测ALDH各亚型在mRNA水平的表达

Trizol法提取HCC-827及HCC-827/GR细胞总RNA，利用反转录试剂盒反转录为cDNA，用qRT-PCR法测定ALDH1A1、ALDH1A3、ALDH1B1、ALDH1L1、ALDH7A1、ALDH3A1、ALDH3A2、ALDH3B1和ALDH3B2 mRNA的表达水平，以管家基因GAPDH为内参。

### 1.3 统计学方法

应用SPSS 11.5统计软件进行分析，计量数据以均数±标准差表示，组间比较采用*t*检验，以*P* < 0.05为差异有统计学意义。

## 2 结果

### 2.1 细胞形态学观察

正常培养条件下，HCC-827细胞形态规整，胞质均匀、清晰，核仁清楚，呈漩涡式排列生长（图 1A）; 采用1.0 μmol/mL吉非替尼培养12周后的HCC-827/GR细胞株，细胞形态呈现不均一，胞质中颗粒增多，细胞排列分散（图 1B）。

**1:**
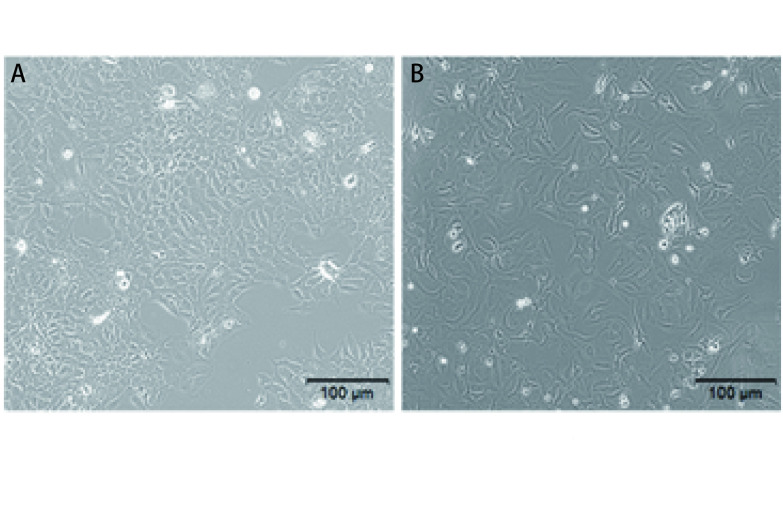
细胞处理前后的形态学改变。 Morphological changes before and after cell treatment.

### 2.2 HCC-827细胞和HCC-827/GR细胞ALDH表达

检测流式细胞术结果显示，人肺腺癌细胞HCC-827中ALDH表达阳性率为39.1%（图 2A），HCC-827/GR细胞ALDH表达阳性率为53.8%（图 2B）。

**2:**
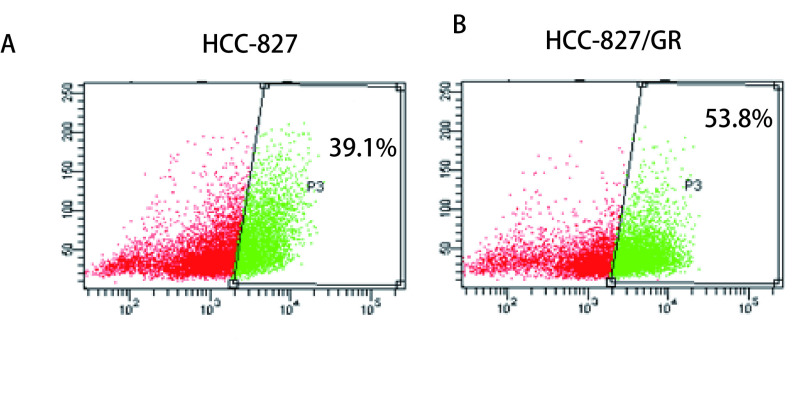
HCC-827与HCC-827/GR细胞ALDH阳性率的的检测结果 The positive ALDH rate of HCC-827 and HCC-827 GR cells

### 2.3 吉非替尼和DEAB对人肺腺癌细胞HCC-827及其耐药株HCC-827/GR的毒性实验

分别在两组细胞中加入1 μmol/mL吉非替尼后，HCC-827细胞的增殖率明显下降，而HCC-827/GR细胞增殖率无变化; 当吉非替尼浓度增加为5 μmol/mL及10 μmol/mL时，两组细胞增殖能力均降低（*P* < 0.05），两组细胞增殖能力的降低呈现为吉非替尼浓度依赖性（图 3A）。分别在两组细胞中加入ALDH抑制剂DEAB100 μmol/L时，HCC-827/GR细胞增殖率无变化; 当DEAB处理浓度增加至300 μmol/L时HCC-827和HCC-827/GR细胞增殖率均受DEAB的剂量影响（图 3B）。

**3:**
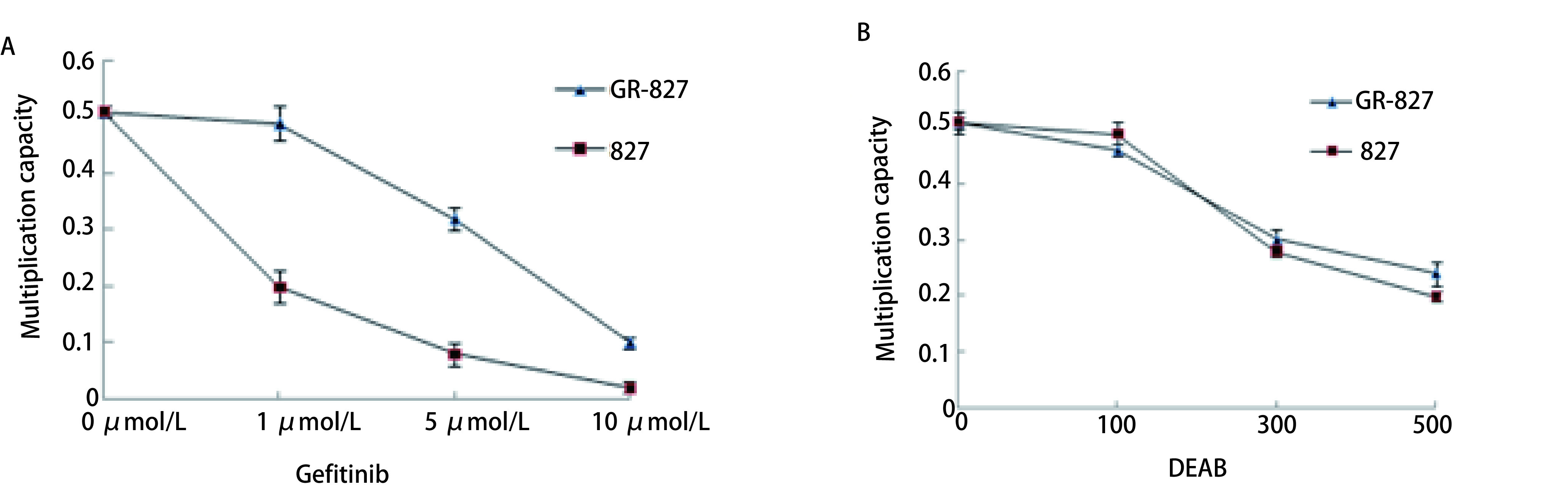
吉非替尼对细胞毒性及耐药性的影响。 Effect of gefitinib on cytotoxicity and drug resistance.

### 2.4 ALDH抑制剂DEAB对HCC-827/GR细胞耐药性的影响

选择无细胞毒性剂量的100 μmol/L DEAB及1 μmol/mL吉非替尼联合处理HCC-827/GR细胞后，MTT结果显示，在DEAB存在的情况下，HCC-827/GR细胞对吉非替尼的敏感性增加，其生长明显受到抑制（*P* < 0.05）（图 4）。

**4:**
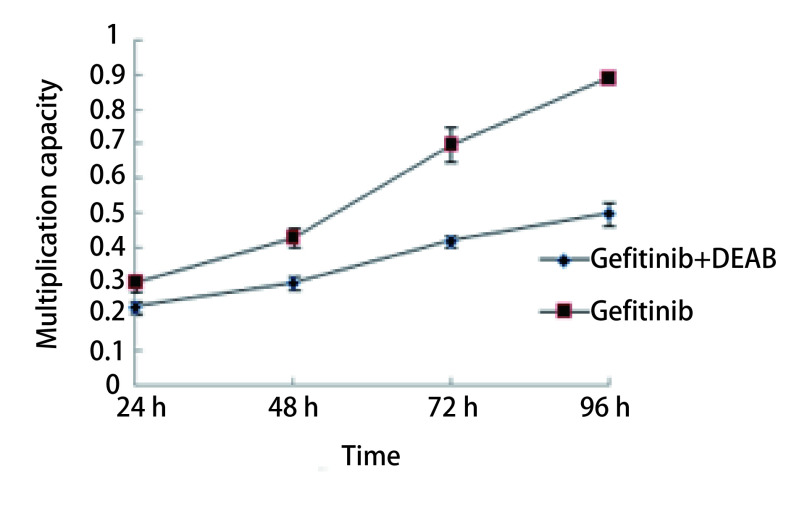
DEAB对HCC-827耐药性的影响 Effect of DEAB on resistance to hcc-827

### 2.5 HCC-827细胞和HCC-827/GR细胞中ALDH各亚型（引物序列见表 1）在mRNA水平的表达

**1:** qRT-PCR引物 qRT-PCR primers

Gene	Primers (5'→3')
*ALDH1A1*	Positive: GCACGCCAGACTTACCTGTC Reverse: CCACTCACTGAATCATGCCA
*ALDH1A3*	Positive: ATCAACTGCTACAACGCCCT Reverse: TATTCGGCCAAAGCGTATTC
*ALDH1B1*	Positive: TGCTGCAGAGTGTCAGCAT Reverse: GGTGGTAGGGTTGACCGTCG
*ALDH1L1*	Positive: ATCTTTGCTGACTGTGA Reverse: GCACCTCTTCTACCACTCTC
*ALDH2*	Positive: TCAAATTACAGGGTCAACTGCT Reverse: GGCTGGGTCTTTACCCTCTC
*ALDH3A1*	Positive: TGTGTCAAAGGCGCCATGAGCAAG Reverse: GGCGTTCCATTCATTCTTGTGCAG
*ALDH3A2*	Positive: CGCTCAACTCTTTCCCATTTG Reverse: TTCCCCAATCCACCTTTGAC
*ALDH3B1*	Positive: ACAAGTCAGCCTTCGAGTCGG Reverse: AGCACCACACAGTTCCCTGC
*ALDH7A1*	Positive: AGGAGAGGTTTGGGAGAAGTCTGT Reverse: TATAAACAGTCGCCTCGCAGTGGT

qRT-PCR结果显示，ALDH1A1、ALDH1L1、ALDH3B1及ALDH7A1在HCC-827/GR细胞中表达量明显高于其在HCC-827细胞中的表达，其中ALDH1A1表达量约为其在HCC-827细胞中表达量的5, 000倍，ALDH1L1表达量约为其在HCC-827细胞中表达量的150倍（*P* < 0.05）; 而ALDH1A3、LDH3A1、ALDH3B2在HCC-827/GR细胞中表达量低于其在HCC-827细胞中的表达，差异均有统计学意义（*P* < 0.05）（图 5）。

**5:**
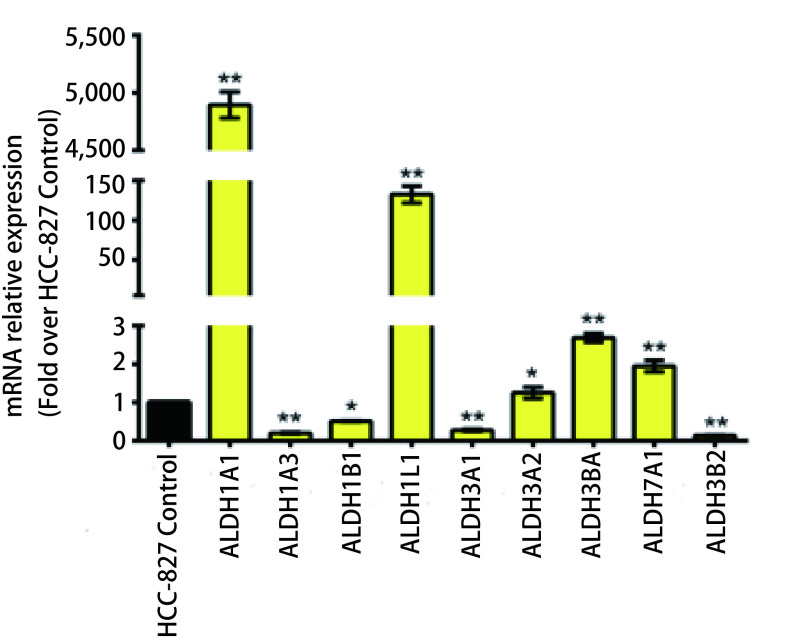
ALDH各亚型在HCC-827细胞和HCC-827/GR细胞中mRNA水平的表达 The expression of mRNA levels in hcc-827 cells and hcc-827/GR cells was expressed in ALDH

## 3 讨论

肺癌已成为全球发病率和死亡率最高的恶性疾病之一，约80%的病例为非小细胞肺癌，肺腺癌为非小细胞肺癌的一种类型。肿瘤的复发及对化疗药物耐受是目前临床治疗肺癌效果不佳的主要原因。分子靶向治疗技术的发展为非小细胞肺癌患者治愈带来了新的曙光。准确寻找分子治疗靶点是成功进行肿瘤靶向治疗的关键^[4]^。

吉非替尼是临床常用治疗非小细胞肺癌的药品，但是很多病人在用药后6个月-12个月内出现耐药^[5]^。耐药是目前临床非小细胞肺癌治疗的棘手问题，肿瘤干细胞是多种肿瘤细胞产生耐药性的重要原因之一，但其机制仍不清楚。研究表明，表皮细胞因子受体突变、PTEN缺失、PI3K点突变等都是非小细胞肺癌发病的可能原因，但是目前仍未寻找到有效解决肿瘤干细胞吉非替尼耐药的策略^[6]^。本实验采用吉非替尼处理人肺腺癌细胞系HCC-827细胞，制备成的HCC-827/GR细胞形态发生了明显的变化，说明吉非替尼治疗可能影响了肿瘤干细胞的生物学特性，导致最终发生耐药。

ALDH是正常干细胞和肿瘤干细胞分离及鉴定的表面标志物之一^[7]^。以往研究证明，ALDH通过对内源性和外源性活性醛的代谢来维持细胞内稳态^[8]^。临床研究发现，高表达ALDH、CD44肺癌患者有较高的复发率^[9]^。本实验发现，ALDH在HCC-827/GR细胞中表达升高，当加入ALDH抑制剂DEAB后HCC-827/GR增殖能力下降。该结果提示，ALDH在维持肺腺癌细胞特性中发挥一定的生物学功能，并且可能参与肺腺癌细胞对吉非替尼耐药过程的形成。该结果与恶性胸膜间质瘤细胞对顺铂耐药的研究结果相似^[10]^。肿瘤细胞针对不同药物产生耐药的机制是多样的，ALDH一方面通过其细胞保护作用对抗醛类、烷类等药物的损伤; 另一方面，它通过视黄酸信号通路调节细胞的更新及分化^[11]^。ALDH有19个家族成员，它们调节了多种细胞功能，包括增殖、分化、生存以及细胞对氧化应激的反应^[12]^，但是关于ALDH各亚型对肺腺癌细胞耐吉非替尼的功能及机制，还没有研究结论。据文献报道，ALDH的多个亚型中9个与肿瘤的发生发展相关^[13, 14]^，本研究检测了这9个基因在肺腺癌细胞HCC-827/GR中的表达，结果显示ALDH1A1、ALDH1L1的表达明显高于HCC-827，提示ALDH1A1、ALDH1L1可能在肺腺癌细胞对吉非替尼耐药过程发挥作用。在后续实验中，采用SiRNA及过表达载体抑制或过表达HCC-827/GR细胞中ALDH1A1、ALDH1L1，通过检测细胞对吉非替尼敏感性变化，进一步明确ALDH1A1、ALDH1L1在肺腺癌细胞吉非替尼耐药过程中的功能。

综上所述，本实验发现ALDH可能参与肺腺癌细胞对吉非替尼耐药过程的形成，其亚型ALDH1A1、ALDH1L1在肺腺癌对吉非替尼耐药过程形成中可能发挥重要的作用。本研究结果将为临床靶向治疗吉非替尼耐药的肺腺癌患者寻找治疗靶点提供理论依据。
